# Effects of Multi-Stage Compression Molding Parameters on Mechanical Properties and Damage Behavior of Cross-Ply Continuous Carbon Fiber-Reinforced Polycarbonate Laminates

**DOI:** 10.3390/polym18212769

**Published:** 2026-11-06

**Authors:** Zhanhui Wang, Haonan Liu, Furen Xiao, Haichao Hu, Yan Bi

**Affiliations:** 1 School of Materials Science and Engineering, Yanshan University, Qinhuangdao 066004, China; 2 School of Mechanical Engineering, Tianjin Sino-German University of Applied Sciences, Tianjin 300350, China

**Keywords:** multi-stage compression molding, carbon fiber, polycarbonate, cross-ply laminates, orthogonal analysis, damage behavior

## Abstract

Processing conditions govern the mechanical response of continuous carbon fiber-reinforced polycarbonate (CCF/PC) laminates. This study examined variations in mechanical properties and associated damage in cross-ply laminates using an L9 orthogonal design varying pre-molding temperature, consolidation pressure, and consolidation time. Tensile, flexural, and short-beam tests were combined with descriptive factor-level mean and range analyses, scanning electron microscopy, and post-test computed tomography. The largest observed factor-level range corresponded to temperature for tensile strength, pressure for tensile modulus, and time for flexural properties and apparent interlaminar shear strength (ILSS). All five properties reached their highest time-level mean at 10 min. Among the tested combinations, 210 °C–1.0 MPa–15 min gave the highest mean tensile strength and apparent ILSS (752 and 21.4 MPa), whereas 210 °C–2.0 MPa–10 min gave the highest tensile modulus, flexural strength, and flexural modulus (61.5 GPa, 720 MPa, and 67.5 GPa). Representative tensile fracture surfaces associated higher specimen strength with matrix attachment and tearing, and lower specimen strength with interfacial separation. Flexural specimens exhibited compression-side damage and delamination. These findings distinguish property-specific processing responses and show that interpreting flexural performance requires consideration of both compression-side and interlaminar damage.

## 1. Introduction

Continuous fiber-reinforced thermoplastic composites (CFRTPs) have attracted sustained attention in aerospace, transportation, and high-performance lightweight structures owing to their high specific strength, high specific stiffness, excellent impact resistance, and weldability, reprocessability, and recyclability in recent years [1,2,3,4,5,6]. Compared with conventional thermoset composites, thermoplastic matrices do not require a chemical curing process, making them more suitable for high-efficiency and short-cycle manufacturing while offering significant advantages in post-service repair, reprocessing, and recycling [2,3,4,5,6,7]. Building upon these advantages, continuous carbon fiber-reinforced thermoplastic laminates have emerged as an important development direction for thermoplastic structural composites, as they combine the high load-bearing capability of continuous fibers with the processing flexibility of thermoplastic matrices [2,3,4,5,6].

Among commonly used thermoplastic resins, polycarbonate (PC) exhibits excellent toughness, dimensional stability, and impact resistance, while requiring relatively lower processing temperatures, making it a promising engineering thermoplastic for structural applications [7,8,9,10,11,12]. Compared with higher-temperature-resistant resins such as polyetheretherketone (PEEK), polyphenylene sulfide (PPS), and polyetherketoneketone (PEKK), PC imposes relatively lower demands on processing equipment and energy consumption. Consequently, it offers unique advantages in continuous fiber-reinforced thermoplastic composite systems where both mechanical performance and manufacturing cost must be considered [8,9,10,11,12]. The interface between PC and carbon fibers is governed not only by mechanical interlocking but also by interfacial molecular interactions, indicating that the interfacial characteristics are jointly influenced by both material characteristics and processing conditions [9]. Studies on the fabrication of CCF/PC laminates using a two-step hot-pressing process have demonstrated that staged consolidation can improve laminate integrity and mechanical performance, highlighting the high sensitivity of PC-based systems to molding routes and consolidation history [10]. Investigations on the in situ consolidation and environmental service behavior of CCF/PC composites have further confirmed that interlaminar bonding quality, interfacial continuity, and internal defects are critical factors governing the final mechanical performance [13,14,15].

Matrix viscosity at processing temperatures affects fiber bundle impregnation, interlayer contact, void elimination, and the establishment of interfacial continuity. Its role varies with polymer chemistry, temperature, and deformation rate, reflecting the different thermal and flow behavior of semicrystalline matrices such as polyamide and polypropylene and amorphous PC [3,4,16,17,18,19,20]. The molding performance of thermoplastic laminates is determined not only by the final consolidation temperature, pressure, and holding time, but also by the pre-consolidation contact condition, thermal history, and compaction history [3,16,17,19,20,21,22]. Thermoplastic composite processing is therefore not a simple heating-and-pressurization operation but rather a multi-stage continuous process involving preheating, interlayer contact, compaction, consolidation, and cooling, during which heat transfer, viscoelastic flow, compaction deformation, and interlaminar slippage collectively govern the final part quality [3,16,17,18,19,20,21,22,23,24]. Consequently, the manufacturing optimization of continuous fiber-reinforced thermoplastic laminates should not rely solely on the adjustment of final consolidation parameters but instead requires systematic investigation of the entire consolidation pathway.

Existing studies have demonstrated that the molding quality of thermoplastic composites is jointly governed by temperature, pressure, interfacial contact conditions, material slippage, and heat transfer behavior [3,5,16]. Investigations into compaction behavior under rapid processing conditions have shown that temperature and loading history can alter the balance between through-thickness compression and in-plane flow, thereby affecting laminate densification and fiber distribution [17]. Moreover, interfacial heat transfer, thermal shrinkage, viscoelastic behavior, and crystallization/melting processes are closely associated with component deformation and residual structural characteristics [18,19,22,23]. These findings indicate that the performance of thermoplastic composites arises from the coupled effects of the processing pathway rather than being determined solely by final processing conditions.

The mechanical performance of thermoplastic laminates is highly sensitive to processing parameters. Studies on PEEK and PPS systems have shown that processing temperature, dwell time, and molding pressure significantly influence flexural, compressive, interlaminar shear, and impact properties, primarily through modifications in void content, resin distribution, fiber compaction, and interfacial bonding, thereby regulating macroscopic mechanical behavior [25,26,27,28,29,30,31,32,33,34]. Research on continuous carbon fiber-reinforced PEEK (CCF/PEEK) has demonstrated that flexural performance varies markedly with processing conditions and correlates with thermal behavior and fracture characteristics [25]. For PPS laminates, thermo-compression and stamping studies have confirmed that fiber re-compaction and resin migration substantially alter interlaminar bonding, affecting static and environmentally conditioned mechanical responses [26,27,28]. Comparative studies of thermoplastic and thermoset unidirectional composites further indicate that interfacial properties, interlaminar toughness, and crack propagation paths critically determine fracture morphology and overall mechanical response [29]. Investigations on PEEK, PPS, and PEKK laminates have additionally revealed that the effects of processing parameters are generally non-monotonic and that structural evolution depends strongly on the specific processing pathway [20,21,30,31,32,33,34]. Collectively, these findings provide a solid foundation for understanding the processing–structure–property relationships in thermoplastic composites.

In comparison with PEEK- and PPS-based systems, studies on continuous carbon fiber-reinforced polycarbonate (CCF/PC) composites remain relatively limited. Nevertheless, existing investigations likewise indicate that their mechanical performance is highly sensitive to processing conditions. Ozaki et al. [11] reported that, for woven carbon fiber/PC laminates, appropriate molding temperature and pressure can produce laminates with minimal voids and strong interfacial adhesion, thereby significantly enhancing flexural strength. Liu et al. [10] fabricated multilayer carbon fiber fabric/PC laminates using a two-step hot-pressing process and demonstrated that staged pressing contributes to improved laminate integrity and mechanical performance. Studies on the in situ consolidation of CCF/PC composites have further shown that interlaminar bonding, void control, and interfacial continuity are critical factors governing final performance [13]. Investigations of the interfacial shear strength between PC and carbon fibers have additionally confirmed the existence of stable interfacial interactions in CCF/PC systems [9]. These findings suggest that high-quality laminate structures cannot be achieved in PC-based composites solely by relying on relatively low processing temperatures; instead, their mechanical performance strongly depends on the regulation of interfacial and interlaminar structures throughout the processing history.

Previous work identifies temperature and loading history, interlayer contact, and compaction as relevant features of thermoplastic laminate processing [3,16,17,19,22,23]. Mechanical studies further connect processing conditions with property-specific responses [11,25,26,27,28,29]. For the present CCF/PC system, the resulting question is how changing the onset of pre-molding pressure, followed by consolidation at a fixed final temperature, affects tensile, flexural, and short-beam responses within one laminate architecture. Fracture and interface studies provide the basis for examining matrix participation, interfacial separation, and internal damage alongside the mechanical results [13,15,20,21,30,31,32,33,34]. Comparing these observations is particularly useful when the processing groups rank differently for strength, stiffness, and short-beam performance.

This study examines the mechanical response and damage behavior of CCF/PC cross-ply laminates manufactured by multi-stage compression molding. Pre-molding temperature determines when the initial 0.4 MPa pressure is applied during heating; consolidation pressure and time are then varied at a fixed final temperature of 240 °C. A three-factor, three-level L9 orthogonal design provides a common experimental basis for comparing tensile strength and modulus, flexural strength and modulus, and short-beam strength. Factor-level means and ranges describe the processing trends, while fracture SEM and post-test CT identify the damage features associated with representative mechanical responses. The analysis connects the elastic and failure responses of the same laminate architecture with interfacial separation, matrix tearing, delamination, and compression-side damage.

## 2. Materials and Methods

### 2.1. Materials

The CCF/PC prepreg tapes used in this study were supplied by Covestro Polymers (China) Co., Ltd. (Shanghai, China), with a nominal thickness of 0.2 mm and a supplier-reported fiber volume fraction of 44%. The reinforcement was TORAY T700S-12K carbon fiber (Toray Industries, Inc., Tokyo, Japan), and the matrix was Makrolon 3107 polycarbonate (PC) supplied by Covestro (Shanghai, China). The basic properties of the constituent materials are summarized in Table 1 and Table 2.

Manufacturer data give a nominal T700S filament diameter of 7 μm and a Makrolon 3107 (Covestro Polymers (China) Co., Ltd., Shanghai, China) melt volume flow rate of 6 cubic centimeters per 10 min at 300 °C under 1.2 kg (ISO 1133-1) [35].

### 2.2. Multi-Stage Compression Molding Process and Experimental Design

**Figure 1:**
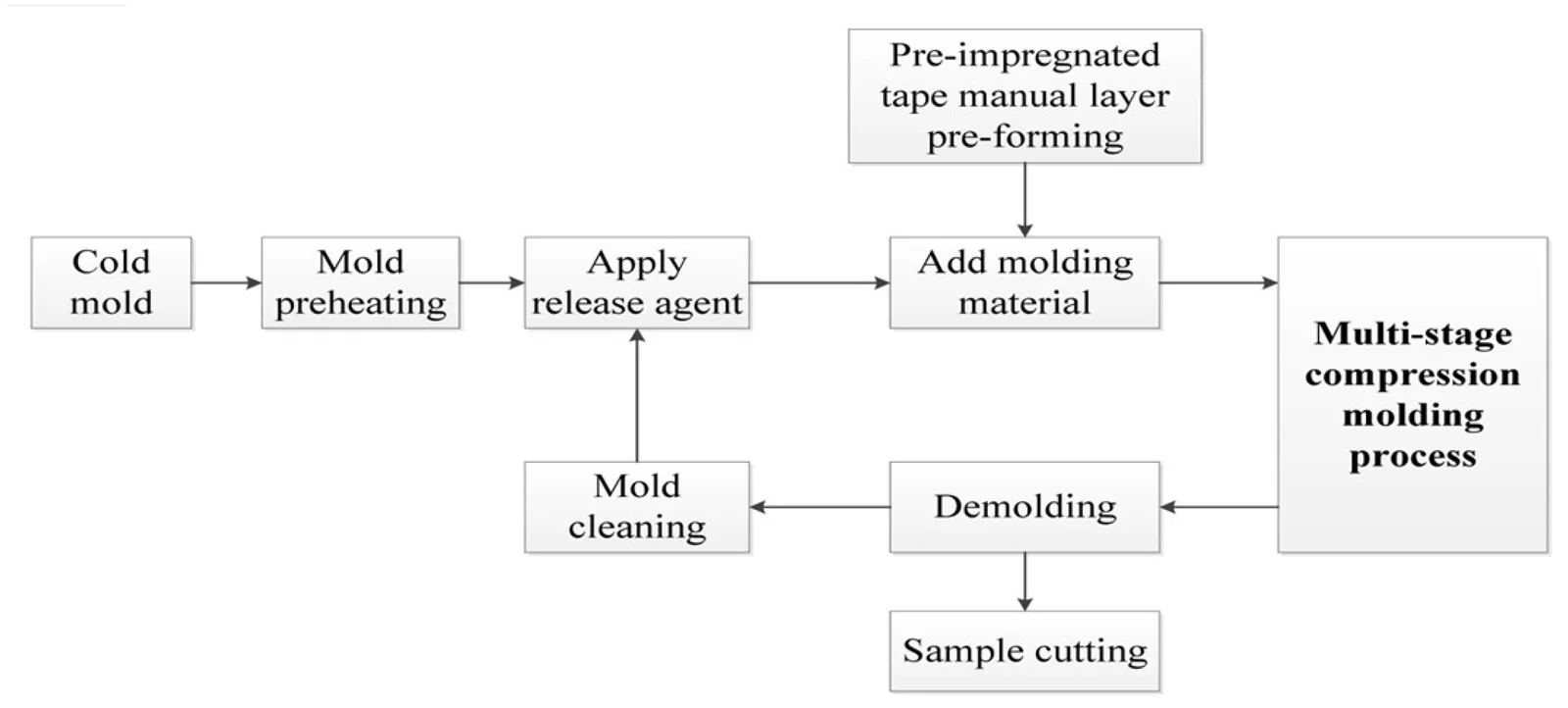
Fabrication process of CCF/PC-laminated composites.

**Figure 2:**
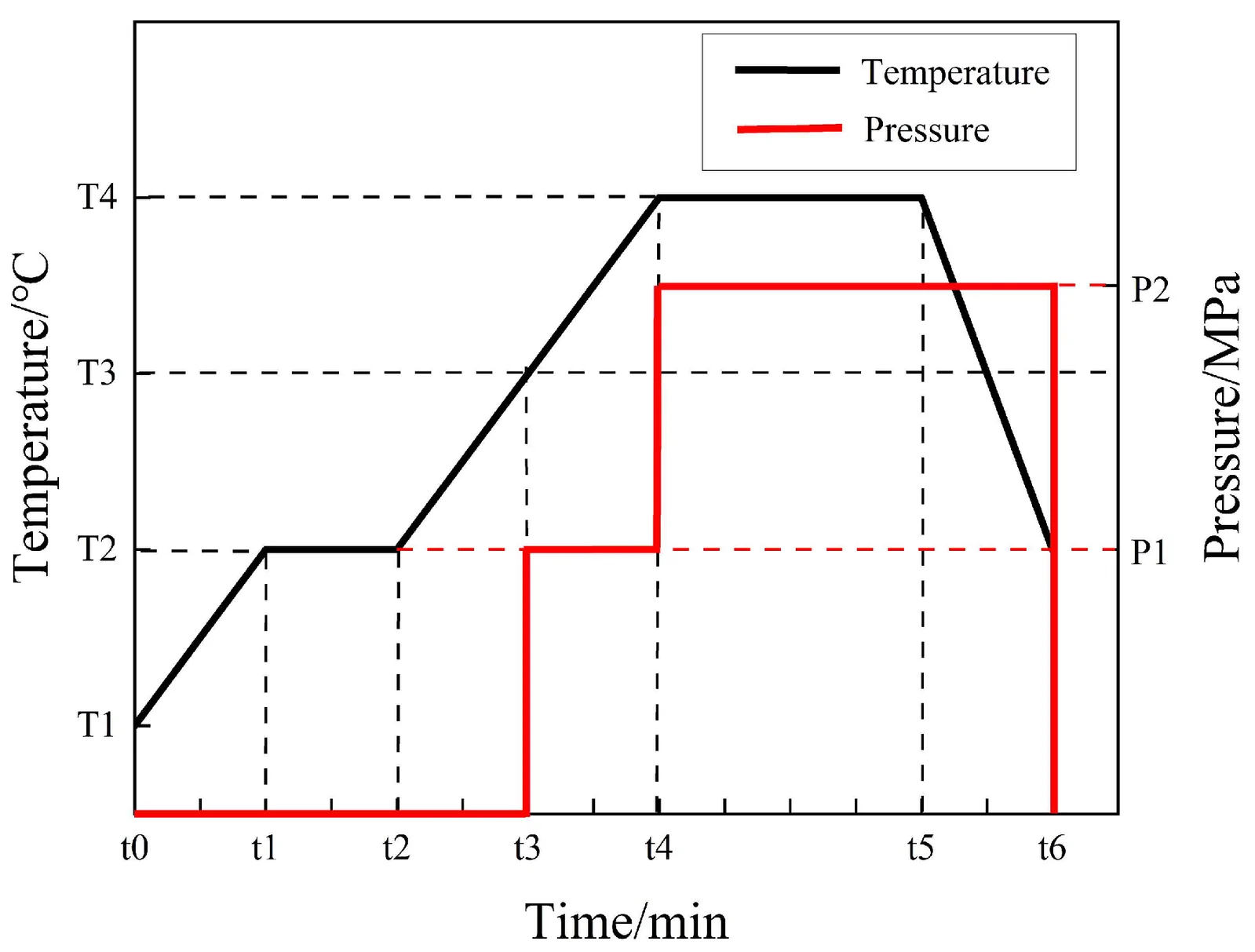
Multi-stage compression molding profile. ($T_{1}$: initial mold temperature; $T_{2}$: mold preheating and demolding temperature; $T_{3}$: pre-molding temperature; $T_{4}$: consolidation molding temperature; $P_{1}$: pre-molding pressure; $P_{2}$: consolidation molding pressure; $t_{3}$: application of pre-molding pressure; $t_{4}$–$t_{5}$: consolidation molding time; $t_{1}$–$t_{6}$: complete molding cycle).

CCF/PC laminates were fabricated using a multi-stage compression molding process. The molding system was jointly developed by Tianjin Tianduan Press Co., Ltd. (Tianjin, China), Shenyang Institute of Automation (Shenyang, China), Chinese Academy of Sciences (Shenyang, China), and Shanghai Chuangke New Materials Technology Co., Ltd. (Shanghai, China). The overall fabrication process is illustrated in Figure 1. The prepreg tapes were stacked in a [0/90]_3s_ lay-up configuration, and the multi-stage compression molding profile is presented in Figure 2. The profile represents one molding cycle for one laminate.

In Figure 2, $T_{1}$ represents the initial mold temperature; $T_{2}$ denotes the mold preheating and demolding temperature; $T_{3}$ is the pre-molding temperature; and $T_{4}$ is the consolidation molding temperature. $P_{1}$ and $P_{2}$ denote pre-molding and consolidation molding pressure, respectively. The interval $t_{4}$–$t_{5}$ represents consolidation molding time, while $t_{1}$–$t_{6}$ spans a complete molding cycle.

Pre-molding temperature, consolidation pressure, and consolidation time were selected as three factors, each at three levels, in a nine-run orthogonal design.

The laminates were fabricated following a standardized molding procedure that had shown good repeatability in routine manufacturing. Each processing parameter combination corresponded to one 500 × 500 mm laminate panel. The 12 plies, each nominally 0.2 mm thick, gave a nominal stacked thickness of 2.4 mm; the closed-mold gap was 2 mm. Mechanical calculations used the measured dimensions of each specimen, rather than assuming an identical final thickness. Five specimens for each mechanical test (tensile, flexural, and short-beam shear) were machined from each panel in accordance with the relevant test standards. The longitudinal axes of all specimens were parallel to the 0° ply direction.

The prepreg was stored in a dry, dark environment at 15–25 °C and a relative humidity not exceeding 40%. Before lay-up, the tapes were placed flat in a hot-air oven and dried at 80–100 °C for 2–4 h; subsequent exposure to air before lay-up was less than 30 min. The mold was heated at 5 °C/min and cooled at 2 °C/min. Consolidation time was counted from the point at which the mold reached 240 °C. Pressure was maintained during cooling and released by unloading the press when the mold reached 80 °C, followed by demolding. All reported processing temperatures refer to the measured mold temperature. Application of 0.4 MPa at 180, 195, or 210 °C during continued heating also changes the duration of the initial low-pressure stage; the temperature factor therefore represents this coupled processing history.

### 2.3. Mechanical Testing

The mechanical properties of the CCF/PC laminates were measured using a Zwick Roell Z250 universal testing machine (ZwickRoell GmbH & Co. KG, Ulm, Germany) at room temperature. Tensile tests followed ASTM D3039/D3039M-17 [36], using specimens with nominal dimensions of 250 × 25 × 2 mm and a crosshead speed of 2 mm/min. Three-point flexural tests followed ASTM D7264/D7264M-15 [37], using nominally 100 × 13 × 2 mm specimens, a support span-to-thickness ratio of 32:1, and a crosshead speed of 1 mm/min. Short-beam tests followed ASTM D2344/D2344M-16 [38], using nominally 12 × 4 × 2 mm specimens, a support span-to-thickness ratio of 4:1, and a crosshead speed of 1 mm/min. Short-beam strength is reported as apparent interlaminar shear strength (ILSS). All tests were conducted under ambient laboratory conditions, and the specimens for each processing group were cut from the corresponding molded panel.

Tensile strain was measured using an extensometer with a 50 mm gauge length. Tensile modulus was calculated by the chord method between 0.1% and 0.3% strain. No bonded end tabs were used; 120-grit abrasive cloth was placed between the hydraulic wedge grips and the specimen, with the abrasive surface facing the specimen. Alignment marks were used to position the specimen along the loading axis. Flexural midspan deflection was measured by an independent contact displacement device. Flexural modulus was calculated over 0.1–0.3% nominal flexural strain using the measured support span and the width and thickness of each specimen.

### 2.4. Thermal and Microstructural Characterization

Differential scanning calorimetry (DSC), thermogravimetric–differential thermal analysis (TG-DTA), scanning electron microscopy (SEM), and industrial X-ray computed tomography (CT) were employed to characterize the thermal response and mass loss during programmed heating, the initial microstructure of the prepreg, and the failure morphology and internal damage features of the specimens. These analyses supported the comparison of processing parameters, mechanical performance, and failure modes.

#### 2.4.1. Differential Scanning Calorimetry (DSC)

Differential scanning calorimetry (DSC) was performed on a 3.625 mg specimen of the as-received prepreg using a NETZSCH DSC 200 F3 instrument (NETZSCH-Gerätebau GmbH, Selb, Germany). The specimen was placed in an aluminum pan with a pierced lid and heated from 30 to 300 °C, then cooled to 30 °C, at 10 °C/min under nitrogen. Heat flow was normalized by the total specimen mass. The characteristic temperatures of the weak thermal response were obtained from the peak evaluation in NETZSCH Proteus. The exported heating and cooling curves are presented without additional smoothing or baseline subtraction; markers on the heating curve indicate the evaluated characteristic temperatures.

The DSC results were evaluated with reference to the general principles of ISO 11357-1 [39].

#### 2.4.2. Thermogravimetric–Differential Thermal Analysis (TG-DTA)

Thermogravimetric–differential thermal analysis (TG-DTA) was performed on a 4.624 mg prepreg specimen under nitrogen using a programmed heating rate of 10 °C/min from 30 to 700 °C. The TG curve was used to determine the retained mass at the consolidation temperature and at the end of the scan. The temperature of maximum mass loss rate was identified from the minimum of the exported derivative thermogravimetric (DTG) signal. The DTA curve was examined alongside TG and DTG to locate thermal features associated with the mass loss region.

A NETZSCH STA 449 F3 Jupiter simultaneous thermal analyzer (Selb, Germany) was used. The TG results were evaluated with reference to the general principles of ISO 11358-1 [40].

#### 2.4.3. Scanning Electron Microscopy (SEM)

Microstructural morphologies were examined using a field-emission scanning electron microscope (FE-SEM, Scios DualBeam, FEI, Hillsboro, OR,USA). Prepreg surfaces and cross-sections were inspected for resin coverage and fiber–matrix distribution. Tensile specimens were examined at their fracture surfaces, and short-beam specimens at delaminated regions, to identify matrix tearing, resin attachment, fiber pull-out, and interfacial separation.

#### 2.4.4. Industrial X-Ray Computed Tomography (CT)

Post-test specimens were examined using a nanoVoxel-1000 series industrial X-ray CT system, with a reconstructed voxel size of 49.4442 μm. Avizo 2025 was used for visualization, image processing, and cross-sectional inspection. Flexural specimens were examined around the midspan loading region; short-beam specimens were examined over their full geometry. The sections were interpreted qualitatively to locate visible interlaminar separations and localized compression-side damage and were linked to the strength of each scanned specimen. The observations characterize the final post-test state.

## 3. Results and Discussion

### 3.1. Experimental Results and Orthogonal Analysis

**Figure 3:**
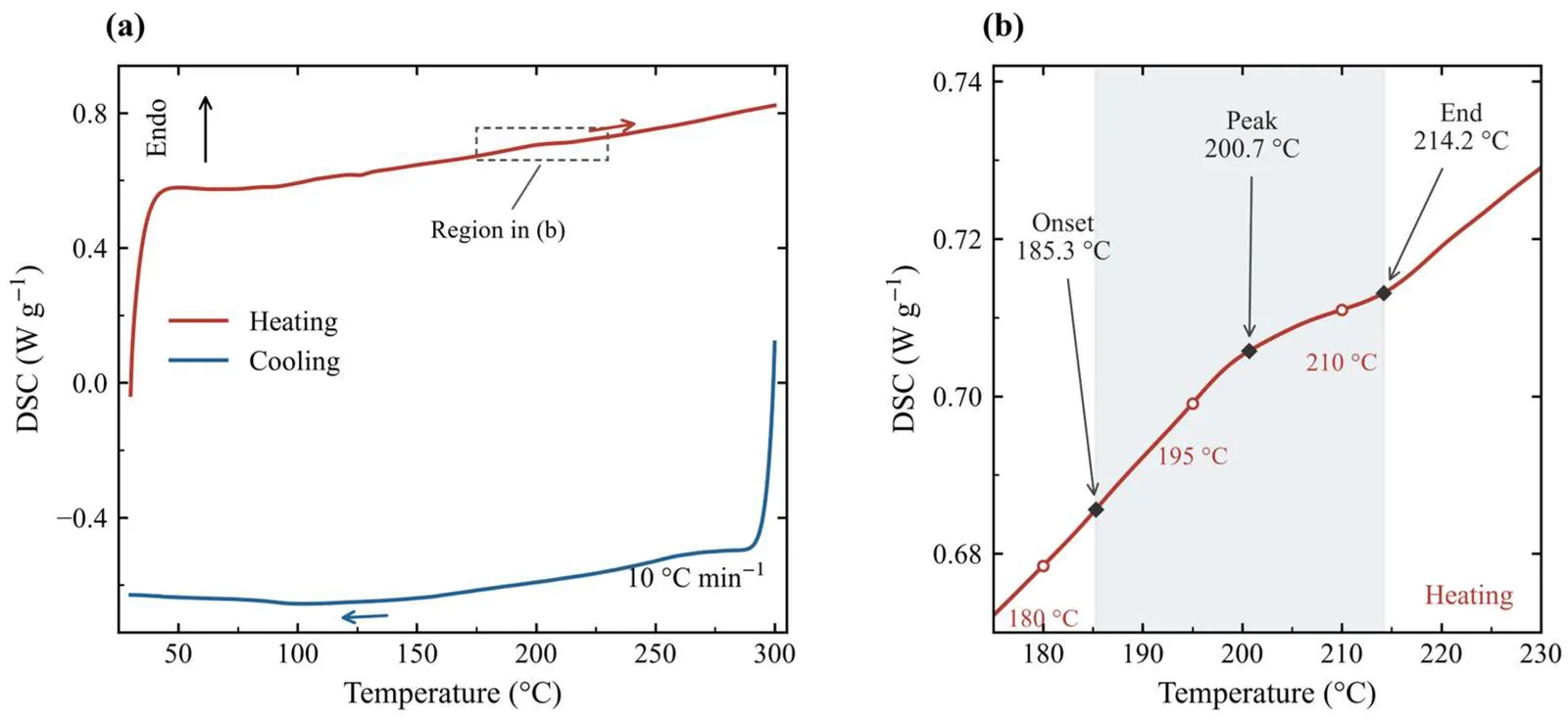
DSC curves of the CCF/PC prepreg: (**a**) heating and cooling curves; (**b**) enlarged heating curve. The curves were plotted from the complete DSC datasets. Diamond symbols indicate the onset, peak, and end temperatures of the weak thermal response; open circles indicate the three pre-molding temperatures. Endothermic heat flow is upward.

The heating curve in Figure 3 exhibits a weak thermal response over approximately 185–215 °C, with evaluated onset, peak, and end temperatures of 185.3, 200.7, and 214.2 °C. The glass transition step of the prepreg was not clearly resolved, so the nominal PC matrix glass transition temperature of 145 °C (Table 2) was used as the temperature reference. All three pre-molding temperatures exceed this reference: 180 °C lies below the onset of the weak response, whereas 195 and 210 °C lie within its interval, on either side of the evaluated peak. The selected levels thus sample different temperatures for the application of initial compaction pressure before consolidation at 240 °C.

**Figure 4:**
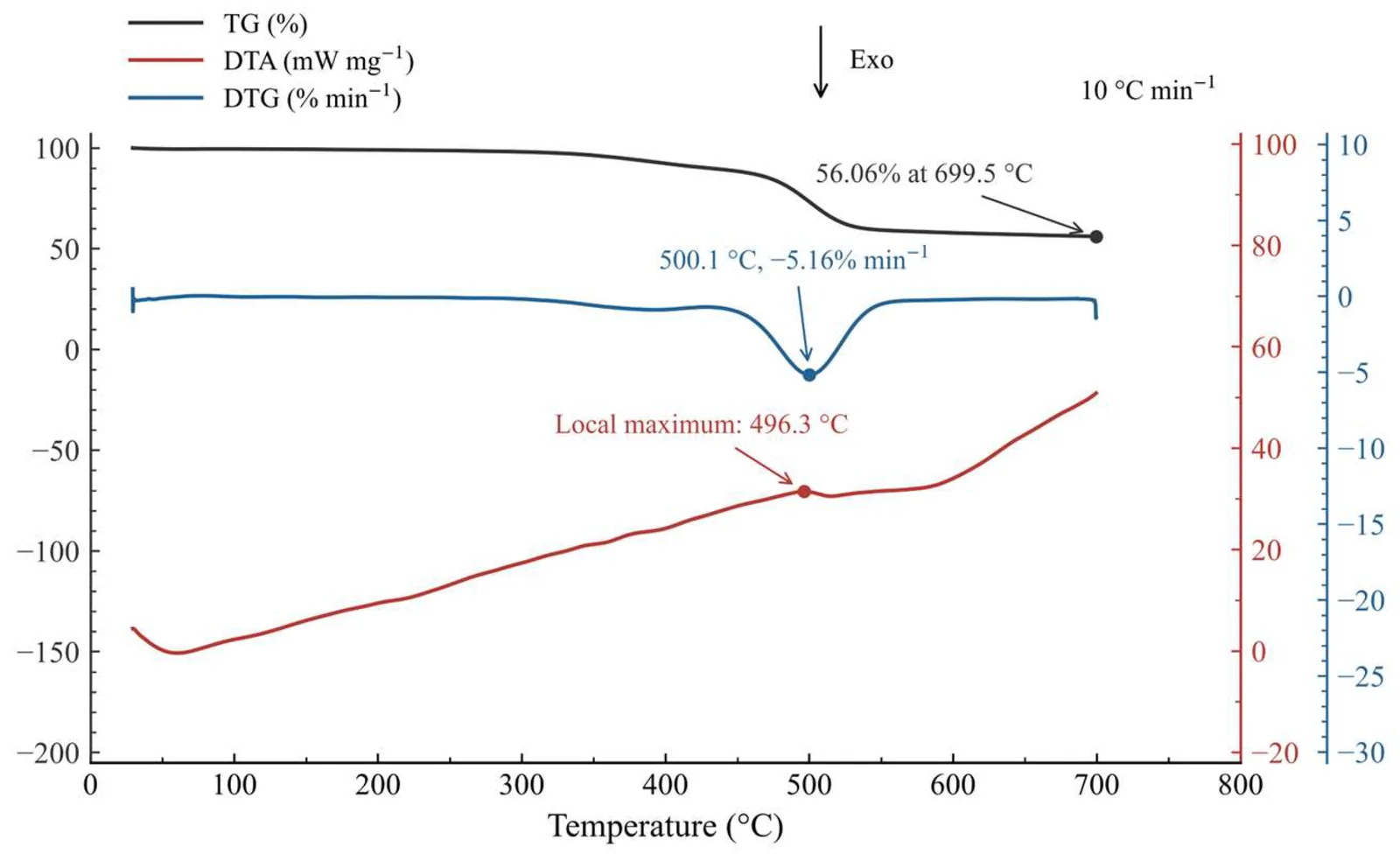
TG, DTA, and DTG curves of the CCF/PC prepreg at a heating rate of 10 °C/min. The markers identify the residual mass at the scan endpoint, the DTG minimum, and the local DTA maximum. The respective ordinate scales apply to each curve; the exothermic direction for DTA is downward.

The TG–DTA curves in Figure 4 show gradual mass loss followed by the principal rapid mass loss stage. The DTG minimum at 500.1 °C corresponds to a maximum mass loss rate of 5.16%/min. A local DTA maximum occurs at 496.3 °C, close to the temperature of this DTG minimum. The retained mass is 98.76% at 240 °C and 56.06% at the scan endpoint of 699.5 °C. Thus, the cumulative mass loss on heating to the consolidation temperature is approximately 1.24%, and the maximum mass loss rate occurs at a substantially higher temperature.

A nine-run, three-factor, three-level orthogonal design was used to examine the effects of pre-molding temperature (A), consolidation pressure (B), and consolidation time (C). The pre-molding temperatures were set at 180, 195, and 210 °C. These levels are above the nominal glass transition temperature of the PC matrix and cover temperatures below the onset and within the weak thermal response interval shown in Figure 3. The consolidation pressure and time levels were selected with reference to the manufacturer-recommended processing conditions and previous work [11]. The factor levels and experimental combinations are presented in Table 3 and Table 4, respectively. The pre-molding pressure and final consolidation temperature were fixed at 0.4 MPa and 240 °C. All groups used the same cross-ply lay-up, allowing processing responses to be compared within a consistent laminate architecture.

The mechanical results for the nine processing groups are summarized in Table 5. Panel means were used in the orthogonal analysis, and the standard deviation and coefficient of variation describe specimen variability within each panel. The descriptive statistics [41] were calculated using Equations (1)–(3).

(1) $$\bar{x}=\frac{1}{n}\sum_{i=1}^{n}x_{i}$$

(2) $$s_{n-1}=\sqrt{\frac{\sum_{i=1}^{n}x_{i}^{2}-n{\bar{x}}^{2}}{n-1}}$$

(3) $$CV=100\times s_{n-1}/\bar{x}$$

In the equations: $\bar{x}$ is the sample mean; $s_{n-1}$ is the sample standard deviation; *CV* denotes the coefficient of variation expressed as a percentage; n represents the number of specimens; and $x_{i}$ is the measured value of the property.

The group-mean properties in Table 5 span 552–752 MPa for tensile strength, 50.2–61.5 GPa for tensile modulus, 396–720 MPa for flexural strength, 39.2–67.5 GPa for flexural modulus, and 11.1–21.4 MPa for apparent ILSS. These results establish the property ranges observed among the tested processing combinations.

Among the tested combinations, Exp.7 (210 °C–1.0 MPa–15 min) had the highest mean tensile strength and apparent ILSS, at 752 and 21.4 MPa. Exp.9 (210 °C–2.0 MPa–10 min) had the highest mean tensile modulus, flexural strength, and flexural modulus, at 61.5 GPa, 720 MPa, and 67.5 GPa. These are rankings of the measured panel means; the following analysis examines the factor-level trends across all nine combinations.

The specimen CV values in Table 5 range from 1.7% to 18.3% for tensile strength, 2.7% to 12.9% for tensile modulus, 3.1% to 23.1% for flexural strength, 1.9% to 11.5% for flexural modulus, and 3.7% to 13.4% for ILSS. Exp.9 combined the highest mean flexural strength and modulus with respective CV values of 3.5% and 1.9%. Exp.8 had the lowest mean flexural strength (396 MPa) and a CV of 23.1%. Reporting both the mean and specimen spread distinguishes the measured performance level from its variation within the tested panel.

**Figure 5:**
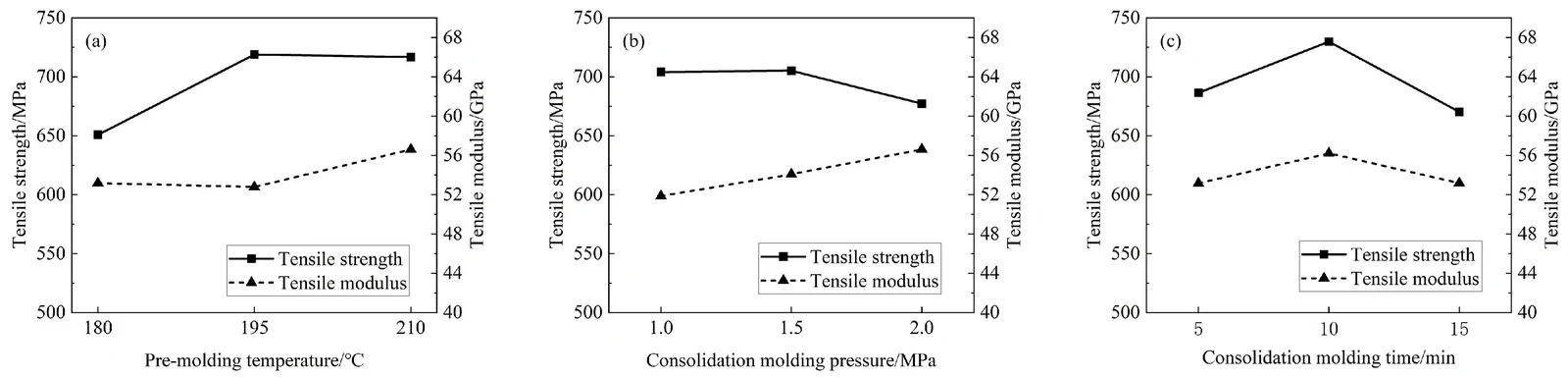
Factor-level means of tensile properties: (**a**) pre-molding temperature; (**b**) consolidation pressure; (**c**) consolidation time. Symbols identify the tested levels; connecting lines guide comparison between levels.

**Figure 6:**
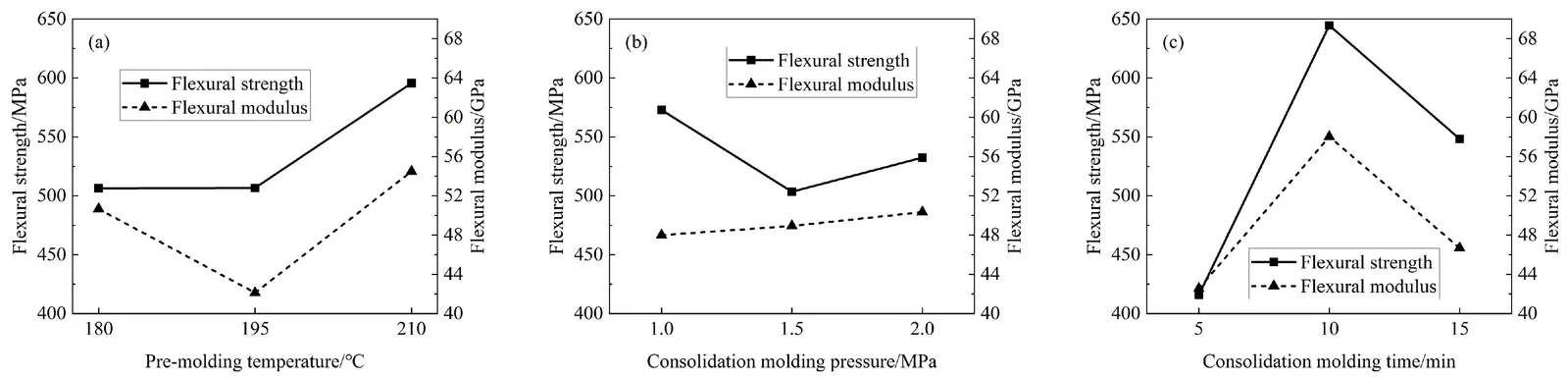
Factor-level means of flexural properties: (**a**) pre-molding temperature; (**b**) consolidation pressure; (**c**) consolidation time. Symbols identify the tested levels; connecting lines guide comparison between levels.

**Figure 7:**
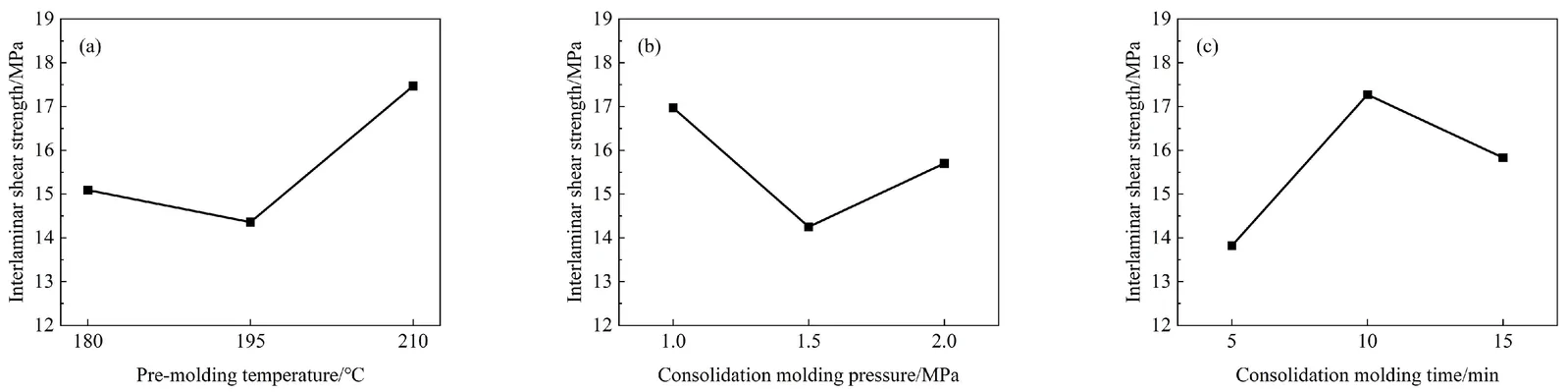
Factor-level means of apparent ILSS: (**a**) pre-molding temperature; (**b**) consolidation pressure; (**c**) consolidation time. Symbols identify the tested levels; connecting lines guide comparison between levels.

To visually analyze the effects of pre-molding temperature, consolidation pressure, and consolidation time on each mechanical property, the mean results of each factor at different levels are summarized in Table 6, and the main-effect trend plots are presented in Figure 5, Figure 6 and Figure 7.

The tensile strength temperature-level means in Figure 5 are 651, 719, and 717 MPa at 180, 195, and 210 °C. The principal change was the increase from 180 to 195 °C; the means at 195 and 210 °C differed by only 2.1 MPa. Pressure-level means were 704, 705, and 677 MPa at 1.0, 1.5, and 2.0 MPa. The time-level mean was highest at 10 min (730 MPa), compared with 686 MPa at 5 min and 670 MPa at 15 min. Since final consolidation remained at 240 °C, the temperature comparison concerns both the thermal state at application of the initial 0.4 MPa pressure and the coupled duration of low-pressure exposure. Differences in matrix deformability at this stage provide a plausible link between the pre-molding temperature and contact development between adjacent tapes.

The tensile modulus pressure-level means increased from 51.9 to 54.1 and 56.6 GPa as pressure increased from 1.0 to 1.5 and 2.0 MPa. Its highest temperature-level and time-level means occurred at 210 °C and 10 min. The opposing locations of the largest modulus mean and smallest strength mean at 2.0 MPa show that the pressure trends of these two tensile properties differ.

For flexural strength, Figure 6 shows time-level means of 416 MPa at 5 min, approximately 645 MPa at 10 min, and 548 MPa at 15 min. The temperature-level means were 506, 507, and 596 MPa, while the pressure-level means were 573, 503, and 532 MPa. These results identify the highest time-level response at 10 min and the highest temperature-level response at 210 °C. The pressure trend was non-monotonic, with the highest mean at 1.0 MPa.

Flexural modulus time-level means were 42.6, 58.0, and 46.7 GPa at 5, 10, and 15 min. Pressure-level means increased from 48.0 to 48.9 and 50.3 GPa, following the same direction as tensile modulus. Improved interply contact is a plausible contribution to this stiffness trend, whereas peak strength also depends on the local distribution of resin and reinforcement. Valverde et al. [16] showed that CF/PPS and CF/PEEK compaction involved both through-thickness compression and in-plane deformation, with temperature-dependent relative contributions. This evidence supports considering material redistribution alongside contact development when interpreting the non-monotonic strength response of the present amorphous-PC laminates.

The short-beam results in Figure 7 show the highest ILSS time-level mean at 10 min, with values of approximately 13.8, 17.3, and 15.8 MPa at 5, 10, and 15 min. The highest temperature-level mean occurred at 210 °C. The pressure-level means were approximately 17.0, 14.3, and 15.7 MPa at 1.0, 1.5, and 2.0 MPa. Both ILSS and flexural strength therefore had their highest pressure-level mean at 1.0 MPa, while their individual group maxima occurred in different processing combinations.

All five properties had their highest time-level mean at 10 min. For flexural strength and modulus, the means increased from 416 MPa and 42.6 GPa at 5 min to 645 MPa and 58.0 GPa at 10 min, then decreased to 548 MPa and 46.7 GPa at 15 min. The increase from 5 to 10 min is consistent with additional time for interply contact development at 240 °C; the lower 15 min means indicate that the benefit did not continue across the full dwell range. Liu et al. [10] used a 240 °C, 6 MPa, 2 min pressing stage to prepare carbon fabric/PC unit laminates, followed by lamination at 210 °C for 3 min. Their route differs in the starting architecture, pressure, and function of each stage, so the dwell response must be interpreted within the specific consolidation route.

The range for each factor was calculated as the difference between its highest and lowest level means, as defined in Equation (4). The results in Table 7 rank the observed main-effect amplitudes within each property and within the investigated factor ranges.

Each level mean gives equal weight to the three panel means at the relevant level. The ranges are descriptive contrasts and are not tests of statistical significance.

(4) $$R_{j}=\max\left({\bar{y}}_{j1},{\bar{y}}_{j2},{\bar{y}}_{j3}\right)-\min\left({\bar{y}}_{j1},{\bar{y}}_{j2},{\bar{y}}_{j3}\right)$$

In Equation (4), $R_{j}$ is the range for factor j, and ${\bar{y}}_{j1}$, ${\bar{y}}_{j2}$, and ${\bar{y}}_{j3}$ are the corresponding means at its three levels. The factor rankings in Table 7 are interpreted alongside the directions of the level mean trends.

The factor-level analysis provides the context for the following comparisons of fracture morphology and the measured strengths of the inspected specimens, with attention to interfacial separation, matrix participation, and compression-side damage.

### 3.2. Prepreg Structure and Tensile Fracture Behavior

The initial resin distribution in the prepreg and the final fracture surfaces provide complementary views of the fiber–matrix assembly. The prepreg images show the material available for contact and redistribution during molding; the tensile fracture images identify whether final separation occurred mainly along fiber surfaces or involved tearing of the surrounding matrix.

**Figure 8:**
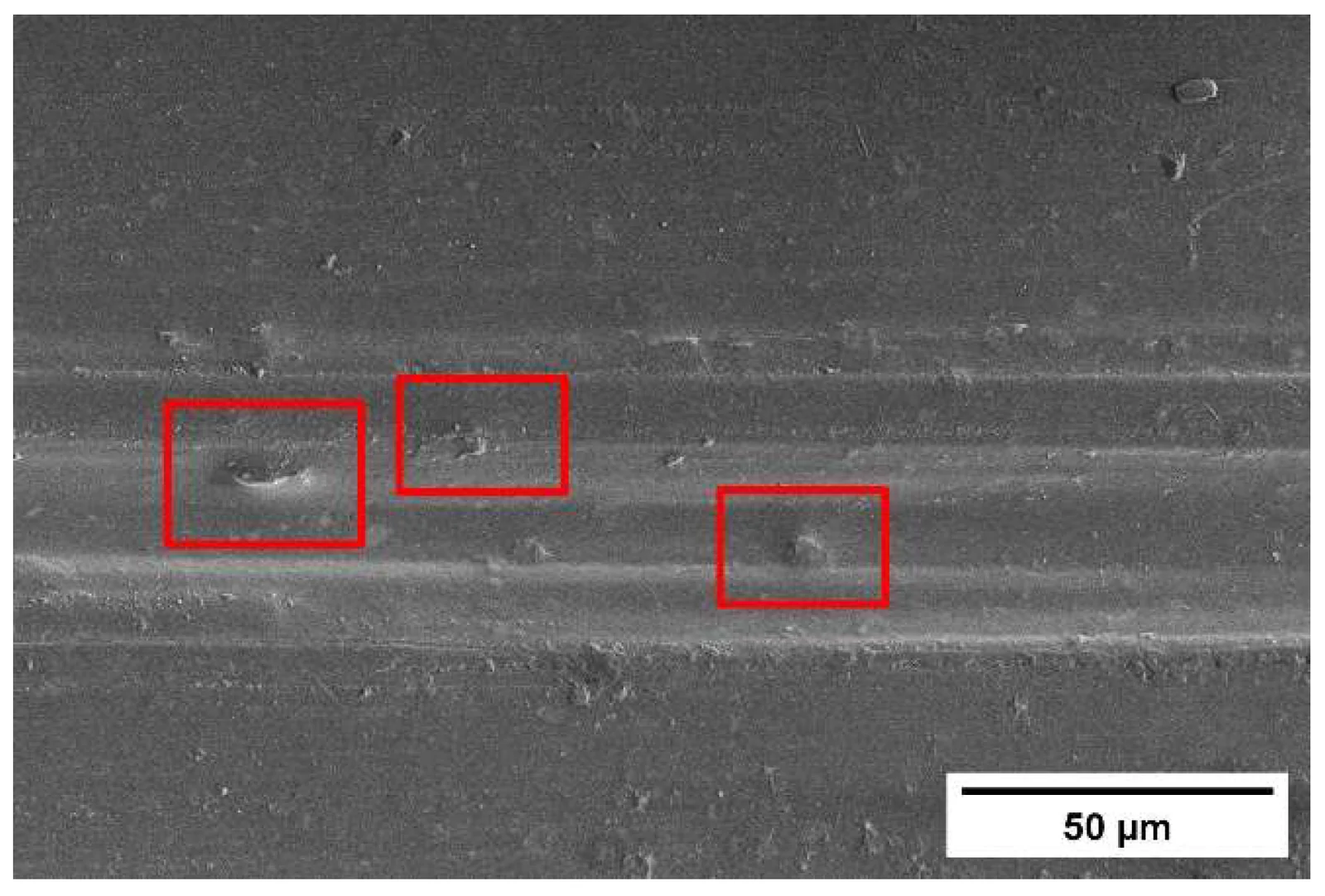
Surface morphology of CCF/PC prepreg tape.

The prepreg surface in Figure 8 was predominantly covered by a continuous resin layer with longitudinal streaks and local resin-rich protrusions. This morphology provides the initial surface condition for contact between adjacent tapes during lay-up and molding.

**Figure 9:**
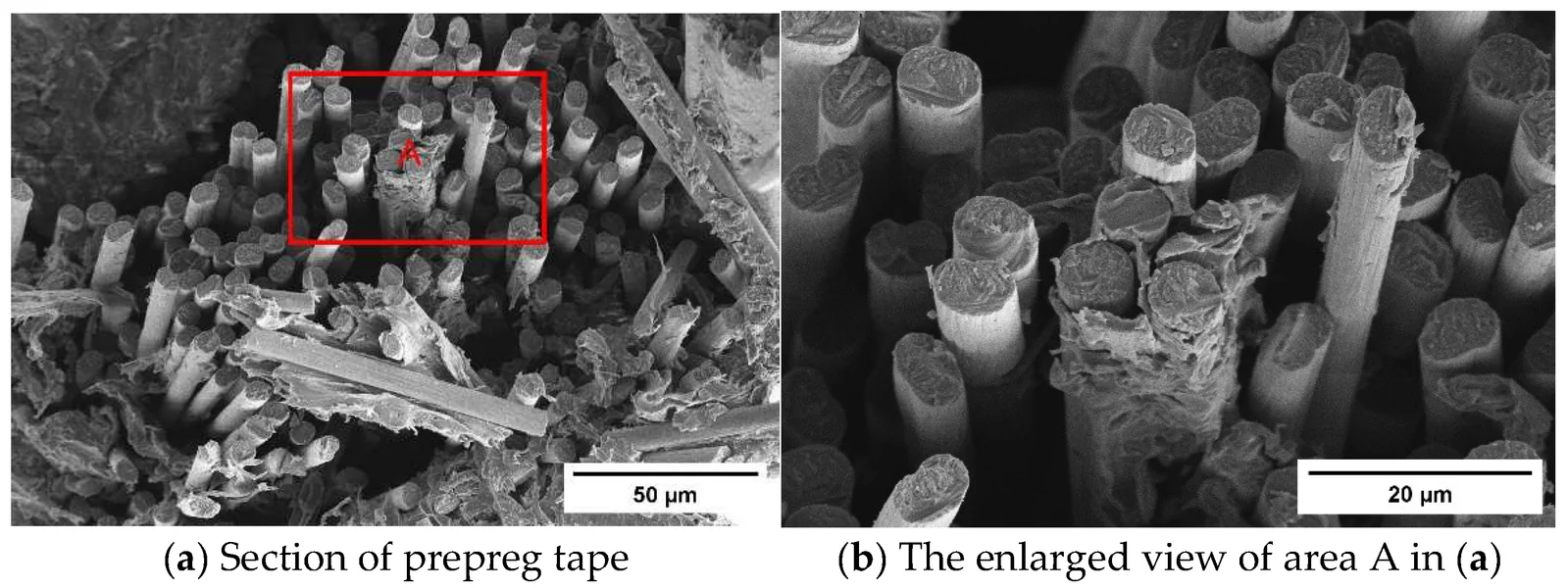
SEM image of carbon fiber distribution of CCF/PC prepreg tape.

The cross-sectional SEM in Figure 9 shows the fiber and resin distribution within the prepreg; Figure 9b is a magnified view of the marked region in Figure 9a. Resin occupied regions within and between fiber bundles, while local gaps and variations in resin distribution remained visible. These observations identify local heterogeneity already present before laminate consolidation and provide a structural reference for the subsequent fracture analysis.

The tensile specimens predominantly exhibited mixed failure, with transverse fracture within the gauge section and edge delamination also observed. In the cross-ply laminate, the 0° plies provide the main fiber-direction tensile load path, while load transfer through the matrix and interfaces couples their response to the transverse 90° plies. Fracture can therefore involve both the fiber-direction load path and separation between neighboring fibers or plies.

The matrix connects adjacent fibers and plies and transfers load between regions with different fiber orientations. Fracture surface matrix attachment and interfacial separation therefore provide a basis for interpreting how tensile failure differs between processing groups.

**Figure 10:**
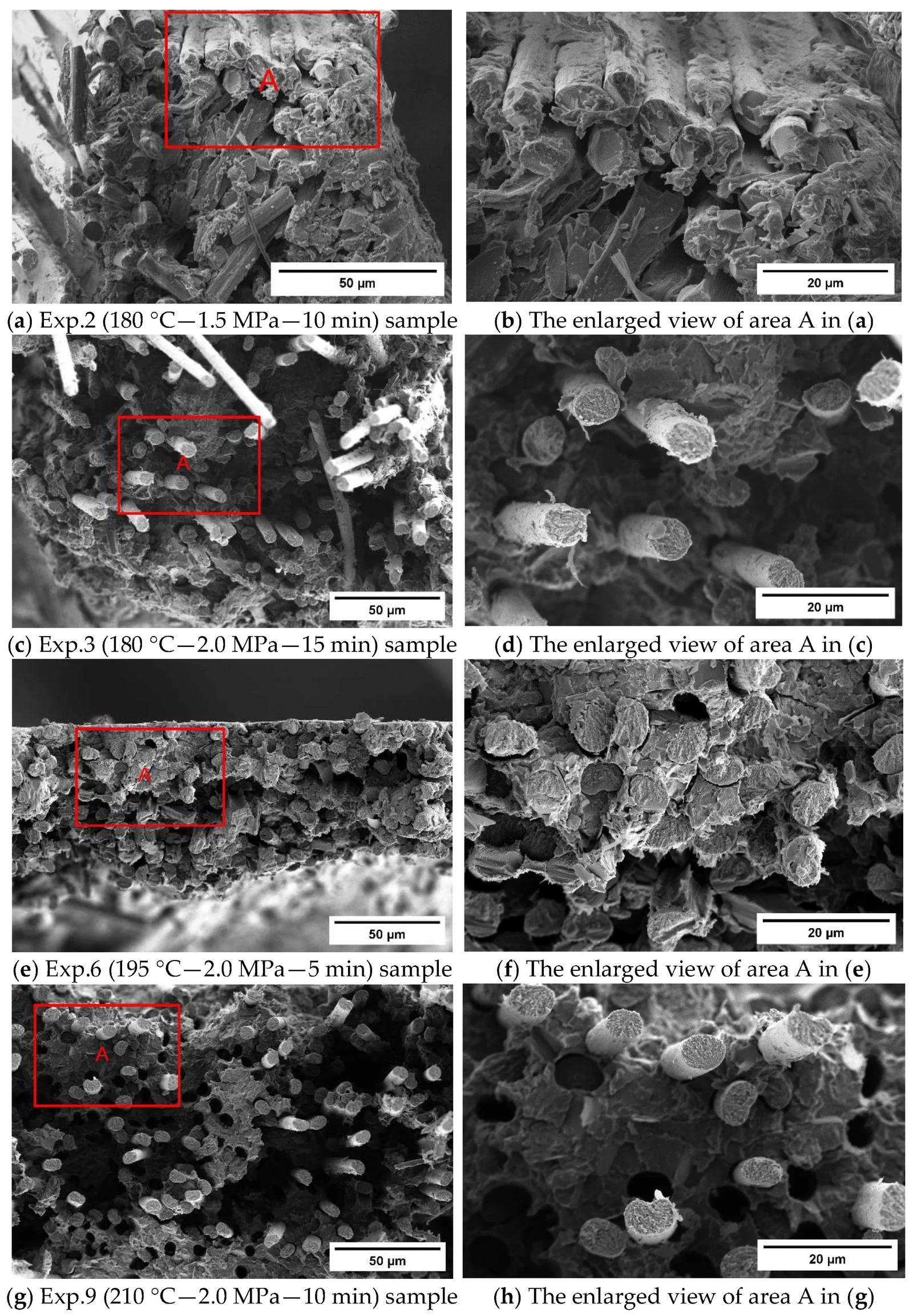
Tensile fracture SEM images: (**a**,**b**) Exp.2, specimen 2, 743 MPa; (**c**,**d**) Exp.3, specimen 3, 418 MPa; (**e**,**f**) Exp.6, specimen 2, 754 MPa; (**g**,**h**) Exp.9, specimen 1, 723 MPa.

The representative tensile fracture SEM images in Figure 10 compare Exp.2 specimen 2 (743 MPa) and Exp.3 specimen 3 (418 MPa) at the same pre-molding temperature. Exp.2 showed matrix tearing and residual resin on fibers, whereas Exp.3 showed more fiber pull-out, separation along bundles and interfaces, and locally exposed fibers. The lower strength of the Exp.3 specimen thus coincided with more interfacial separation; the higher strength of the Exp.2 specimen coincided with greater matrix participation in fracture.

In Exp.3, separation along bundles and fiber surfaces is consistent with disruption of load transfer between neighboring fibers. Matrix tearing and resin attachment in Exp.2 indicate that fracture also passed through the surrounding polymer. The 743 versus 418 MPa specimen strength contrast therefore accompanies different fracture paths within the fiber–matrix assembly.

Exp.6 specimen 2 also showed matrix tearing and reached a tensile strength of 754 MPa. The occurrence of matrix tearing in both Exp.2 and Exp.6 extends this fracture observation to two different processing combinations with relatively high specimen tensile strengths.

Exp.9 specimen 1 had a tensile strength of 723 MPa and showed protruding fibers and pull-out cavities together with resin attached to portions of the fibers and their roots. Its fracture surface therefore contains both interfacial separation and matrix remnants.

Kore et al. [42] reported favorable fiber–PC wettability for epoxy-sized textile-grade carbon fiber/PC composites using fracture SEM together with independent interface characterization. Their work supports considering resin attachment alongside fiber exposure in fracture interpretation. In the present images, the matrix tearing in Exp.2 and Exp.6, interfacial separation in Exp.3, and coexistence of pull-out and resin attachment in Exp.9 distinguish the observed fracture paths. These morphological comparisons associate tensile strength with the balance between matrix and interfacial fracture rather than with fiber pull-out alone.

The tensile SEM observations associate the lower strength of the inspected Exp.3 specimen with separation-dominated fracture, while the inspected Exp.2 and Exp.6 specimens combine higher strengths with matrix tearing. This specimen-specific evidence complements the factor-level analysis by identifying the material regions involved in final separation.

At the same consolidation pressure of 2.0 MPa, Exp.6 and Exp.9 had tensile moduli of 55.9 and 61.5 GPa, whereas their tensile strength means were close at 744 and 736 MPa. Both combinations therefore produced relatively high mean tensile strengths with different initial elastic responses.

### 3.3. Flexural and Short-Beam Responses in Relation to Damage Morphology

The flexural and short-beam tests probe different combinations of tensile, compressive, and shear response. In accordance with the short-beam method described above, the measured strength is reported as apparent ILSS and interpreted together with the fracture morphology. The comparison below first examines interlaminar separation and matrix fracture under short-beam loading, then relates them to the additional compression-side damage observed after flexural testing.

Flexural failure included interlaminar shear failure between the loading nose and a support, compression failure near the loading point, and mixed failure. The post-test CT sections distinguish internal interlaminar separations from localized compression-side damage within these specimens.

The tested flexural specimens remained connected after loading. Accordingly, the CT analysis focuses on internal separations and localized damage within the retained specimen geometry.

**Figure 11:**
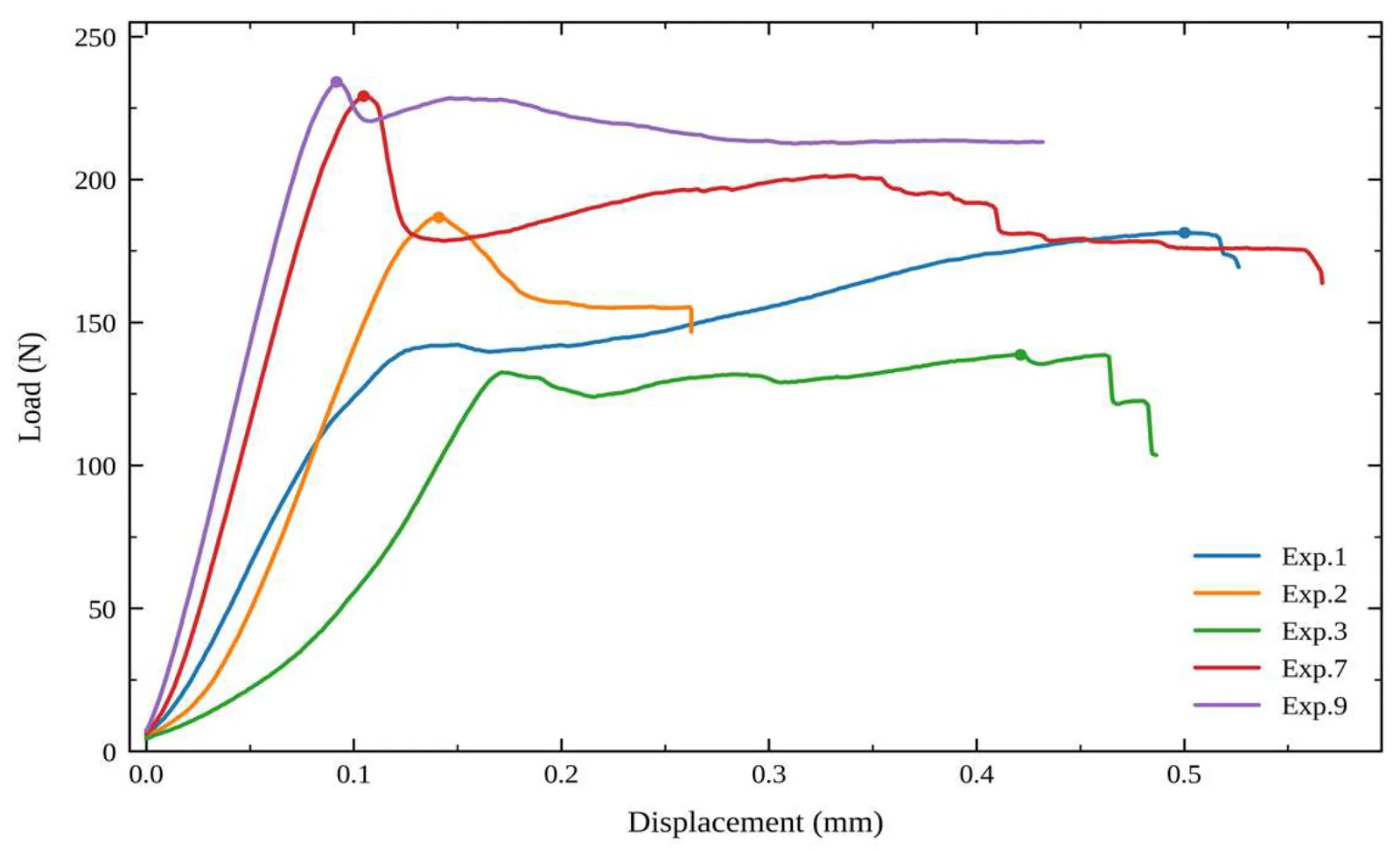
Representative short-beam load–displacement curves for Exp.1, Exp.2, Exp.3, Exp.7, and Exp.9. Displacement was independently measured; circular markers identify the maximum loads.

Representative short-beam load–displacement curves for Exp.1, Exp.2, Exp.3, Exp.7, and Exp.9 are shown in Figure 11. The curves differ in maximum load, displacement at maximum load, and subsequent load retention; some exhibit load recovery after an earlier drop. All valid short-beam specimens included in the analysis were classified as interlaminar shear failures. Together with the post-test interlaminar separations and fracture surface observations, these results support interlaminar shear as the dominant classified failure mode. The peak-load-based strength is reported as apparent ILSS, reflecting the complex short-beam stress state.

**Figure 12:**
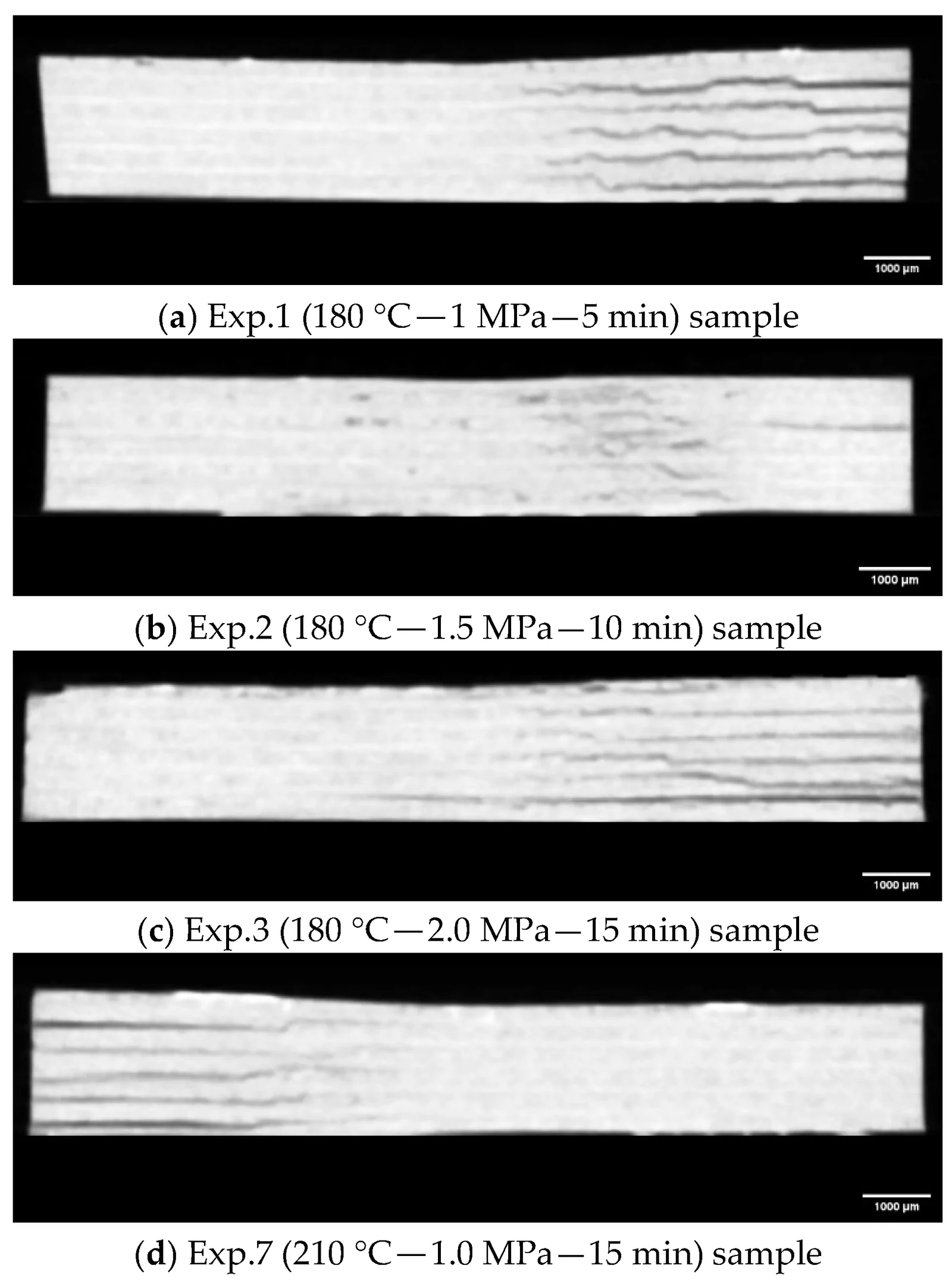
Post-test CT images of short-beam specimens: (**a**) Exp.1, specimen 2, 17.7 MPa; (**b**) Exp.2, specimen 3, 19.5 MPa; (**c**) Exp.3, specimen 2, 11.4 MPa; (**d**) Exp.7, specimen 3, 21.1 MPa. Strengths are the apparent ILSS of the scanned specimens.

**Figure 13:**
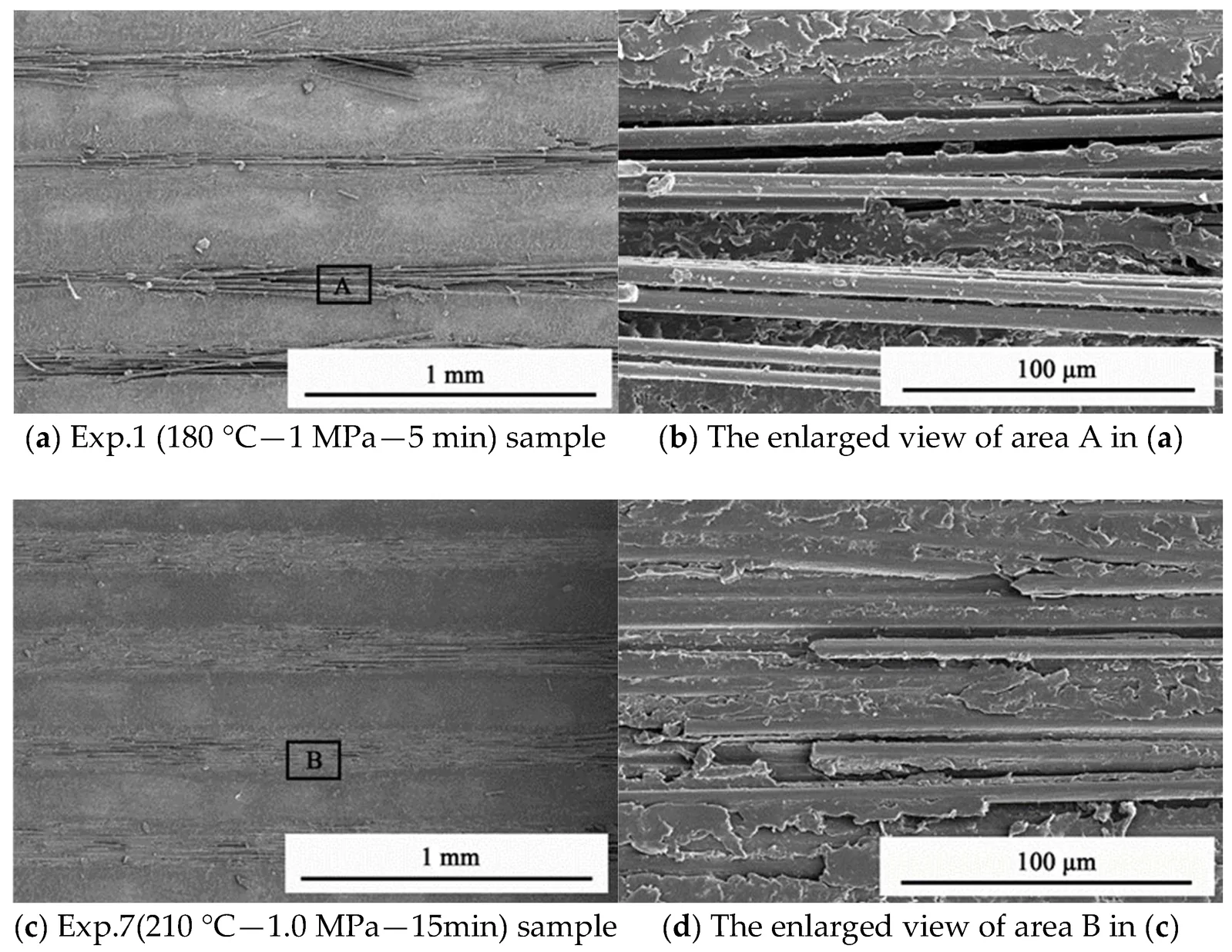
Short-beam fracture SEM images: (**a**,**b**) Exp.1, specimen 1, apparent ILSS 13.8 MPa; (**c**,**d**) Exp.7, specimen 3, apparent ILSS 21.1 MPa.

The short-beam CT images in Figure 12 show Exp.1 specimen 2 (17.7 MPa) and Exp.3 specimen 2 (11.4 MPa), both with elongated interlaminar separations extending inward from an end region. The SEM images of Exp.1 in Figure 13a,b were obtained from a different coupon, specimen 1 (13.8 MPa), and show delamination steps and exposed fibers with limited resin attachment. These observations identify interlaminar and interfacial separation at different observation scales.

The short-beam CT sections in Figure 12 also show localized internal cracks in Exp.2 specimen 3 (19.5 MPa) and several separations near an end region in Exp.7 specimen 3 (21.1 MPa). The higher measured strength of the latter specimen coexisted with visible post-test end-region delamination. These post-test sections locate final separations, while the SEM comparison identifies the fracture features within the separated material.

In Figure 13c,d, Exp.7 specimen 3 (21.1 MPa) showed resin retained on fibers and matrix tearing, whereas Exp.1 specimen 1 (13.8 MPa) showed more exposed fibers and delamination steps. The higher-strength inspected specimen thus exhibited greater matrix participation in fracture. The CT and SEM observations for Exp.7 were obtained from the same short-beam specimen.

**Figure 14:**
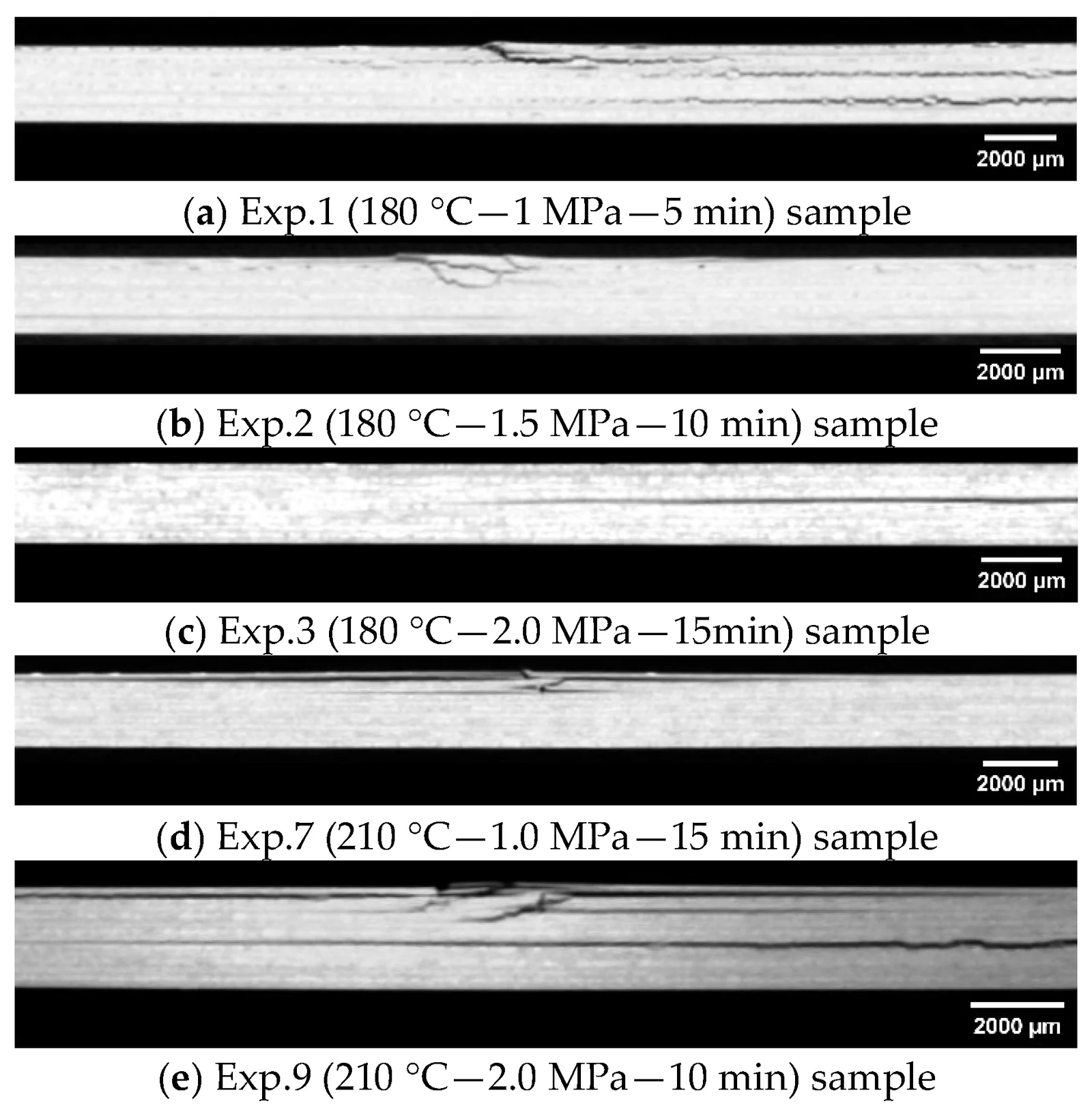
Post-test flexural CT images: (**a**) Exp.1, specimen 2, 349 MPa; (**b**) Exp.2, specimen 2, 613 MPa; (**c**) Exp.3, specimen 2, 455 MPa; (**d**) Exp.7, specimen 1, 668 MPa; (**e**) Exp.9, specimen 1, 744 MPa. Strengths correspond to the scanned specimens.

The flexural CT sections in Figure 14 distinguish interlaminar separations from localized compression-side damage. Exp.1 specimen 2 (349 MPa) showed elongated interlaminar separations. Exp.2 specimen 2 (613 MPa) showed a more evident localized compression-side damage region, whereas Exp.3 specimen 2 (455 MPa) exhibited a different distribution of localized and interlaminar features. The sections locate the material regions involved in the final bending failure of these individual specimens.

Exp.7 flexural specimen 1 (668 MPa) showed localized compression-side damage without a prominent continuous delamination band. Exp.9 flexural specimen 1 (744 MPa) showed both compression-side damage and interlaminar separation. The coexistence of a higher flexural strength with both damage features emphasizes the need to distinguish final damage morphology from the load capacity reached before failure. Ozaki et al. [11] related the flexural strength of textile carbon fiber/PC laminates to molding quality and discussed compression-side fiber microbuckling. This provides a mechanical basis for considering matrix support of compressed fibers alongside interlaminar load transfer. The present CT evidence supports this regional interpretation of bending damage without resolving individual fiber microbuckling.

Exp.2 and Exp.4 provide a further comparison at a common 10 min dwell: their mean flexural strengths were close at 609 and 606 MPa, but their mean flexural moduli were 61.8 and 44.8 GPa. Since modulus is determined from the initial load–deflection response and strength from the peak load, the measured difference is concentrated in the initial stiffness rather than the peak nominal stress. Both pre-molding temperature and consolidation pressure differed between these groups.

Both flexural moduli were calculated from independently measured midspan deflection over the same nominal strain interval.

The results identify two processing trends within the investigated ranges: a 10 min time-level maximum shared by all five properties, and increasing modulus means with pressure without a corresponding monotonic strength increase. The representative fracture comparisons add a structural interpretation at the specimen level. Tensile and short-beam strength differences coincide with changes in matrix tearing and interfacial separation, while bending additionally involves localized compression-side damage. The factor-level trends and fracture observations therefore describe complementary aspects of the response of this cross-ply CCF/PC system.

## 4. Conclusions

The processing response of CCF/PC cross-ply laminates was examined using an L9 orthogonal design, mechanical testing, thermal characterization, SEM, and post-test CT. The following conclusions apply to the investigated laminate architecture and process levels.

1. The largest descriptive factor-level range corresponded to pre-molding temperature for tensile strength, consolidation pressure for tensile modulus, and consolidation time for flexural strength, flexural modulus, and apparent ILSS. These property-dependent rankings identify which investigated factor showed the largest observed variation for each response.
2. All five properties had their highest time-level mean at 10 min, while the tested combinations producing the highest individual panel means depended on the property. Increasing pressure raised both modulus means without a monotonic strength increase. Initial elastic load transfer and failure resistance therefore exhibit distinct processing responses.
3. The inspected tensile specimens linked separation-dominated fracture to lower strength and matrix tearing to higher strength. The coexistence of fiber pull-out and resin attachment in another high-strength specimen further shows that the fracture path through the fiber–matrix assembly, rather than any single surface feature, is relevant to interpreting tensile performance.
4. All valid short-beam specimens were classified as interlaminar shear failures, with the inspected Exp.7 specimen showing more matrix tearing and resin retention than the Exp.1 specimen. Under flexural loading, the inspected Exp.7 specimen exhibited localized compression-side damage without a prominent continuous delamination band, whereas the higher-strength Exp.9 specimen showed both compression-side damage and interlaminar separation. These observations distinguish matrix/interfacial fracture under short-beam loading from combined compression-side and interlaminar damage under flexural loading, providing a structural context for the different strength responses.

## Figures and Tables

**Table 1:** Basic performance parameters of continuous carbon fiber (CCF).

Grade	Tow	Tensile Strength/MPa	Tensile Modulus/GPa	Elongation/%
T700S	12K	4900	230	2.1

**Table 2:** Basic performance parameters of polycarbonate (PC).

Grade	Stress at Break/MPa	Yield Stress/MPa	Glass Transition Temperature/℃
Makrolon 3107	70	66	145

**Table 3:** Factors and levels of orthogonal experiment.

Level	Factor		
	Pre-Molding Temperature (A)/°C	Consolidation Molding Pressure (B)/MPa	Consolidation Molding Time (C)/min
1	180	1.0	5
2	195	1.5	10
3	210	2.0	15

**Table 4:** Plan of orthogonal experiment.

Experiment Number	Factor		
	A/°C	B/MPa	C/min
1	1 (180)	1 (1.0)	1 (5)
2	1	2 (1.5)	2 (10)
3	1	3 (2.0)	3 (15)
4	2 (195)	1	2
5	2	2	3
6	2	3	1
7	3 (210)	1	3
8	3	2	1
9	3	3	2

**Table 5:** Test results of mechanical properties of CCF/PC-laminated composites.

Experiment Number	Factor			Result									
	A/℃	B/MPa	C/min	Tensile Strength		Tensile Modulus		Flexural Strength		**Flexural Modulus**		**Apparent ILSS**	
				$\sigma_{t}$/MPa	CV/%	$E_{t}$/GPa	CV/%	$\sigma_{f}$/MPa	CV/%	$E_{f}$ **/GPa**	**CV/%**	**ILSS/MPa**	**CV/%**
1	180	1.0	5	654 ± 92	14.0	50.2 ± 4.0	8.0	442 ± 92	20.8	45.9 ± 1.0	2.3	15.4 ± 2.1	13.4
2	180	1.5	10	747 ± 61	8.2	56.8 ± 4.3	7.6	609 ± 29	4.7	61.8 ± 1.9	3.1	17.8 ± 1.8	9.9
3	180	2.0	15	552 ± 101	18.3	52.5 ± 6.0	11.5	469 ± 70	14.9	44.4 ± 3.6	8.0	12.2 ± 0.8	6.7
4	195	1.0	10	706 ± 51	7.2	50.4 ± 6.5	12.9	606 ± 49	8.1	44.8 ± 4.0	8.9	14.1 ± 0.8	5.9
5	195	1.5	15	707 ± 44	6.2	52.1 ± 4.4	8.4	505 ± 62	12.4	42.4 ± 4.9	11.5	13.9 ± 0.5	3.7
6	195	2.0	5	744 ± 28	3.8	55.9 ± 1.5	2.7	409 ± 36	8.9	39.2 ± 2.2	5.7	15.0 ± 1.2	7.8
7	210	1.0	15	752 ± 65	8.6	55.0 ± 3.2	5.9	671 ± 21	3.1	53.3 ± 1.5	2.8	21.4 ± 1.3	6.0
8	210	1.5	5	662 ± 42	6.3	53.4 ± 5.1	9.5	396 ± 91	23.1	42.7 ± 3.8	9.0	11.1 ± 0.6	5.3
9	210	2.0	10	736 ± 13	1.7	61.5 ± 3.9	6.3	720 ± 25	3.5	67.5 ± 1.3	1.9	19.9 ± 1.2	6.0

Values are mean ± sample standard deviation. Displayed values are rounded; calculations use the unrounded records.

**Table 6:** Analysis results of the mean mechanical properties of CCF/PC-laminated composites under different levels of various factors.

Experimental Indicators	Factor	Level 1	Level 2	Level 3
Tensile strength ($\sigma_{t}$)/MPa	A	651	719	717
	B	704	705	677
	C	686	730	670
Tensile modulus ($E_{t}$)/GPa	A	53.2	52.8	56.6
	B	51.9	54.1	56.6
	C	53.2	56.2	53.2
Flexural strength ($\sigma_{f}$)/MPa	A	506	507	596
	B	573	503	532
	C	416	645	548
Flexural modulus ($E_{f}$)/GPa	A	50.7	42.1	54.5
	B	48.0	48.9	50.3
	C	42.6	58.0	46.7
Apparent ILSS/MPa	A	15.1	14.4	17.5
	B	17.0	14.3	15.7
	C	13.8	17.3	15.8

**Table 7:** Range analysis of mechanical properties of CCF/PC-laminated composites.

Experimental Indicators	Factor	Range (R)	Range Order	Level with Highest Mean
Tensile strength ($\sigma_{t}$)/MPa	A	68.1	A>C>B	A2
	B	28.1		B2
	C	59.5		C2
Tensile modulus ($E_{t}$)/GPa	A	3.8	B>A>C	A3
	B	4.8		B3
	C	3.1		C2
Flexural strength ($\sigma_{f}$)/MPa	A	89.3	C>A>B	A3
	B	69.5		B1
	C	229		C2
Flexural modulus ($E_{f}$)/GPa	A	12.4	C>A>B	A3
	B	2.3		B3
	C	15.5		C2
Apparent ILSS/MPa	A	3.1	C>A>B	A3
	B	2.7		B1
	C	3.5		C2

## Data Availability

The data presented in this study are available on request from the corresponding author.
